# Supplementary figures and images for: Resolving drug selection and migration in an inbred South American Plasmodium falciparum population with identity-by-descent analysis
Source: PLoS Pathog. 2022 Dec 21;18(12):e1010993. doi: 10.1371/journal.ppat.1010993 (PMC9815574; doi:10.1371/journal.ppat.1010993)

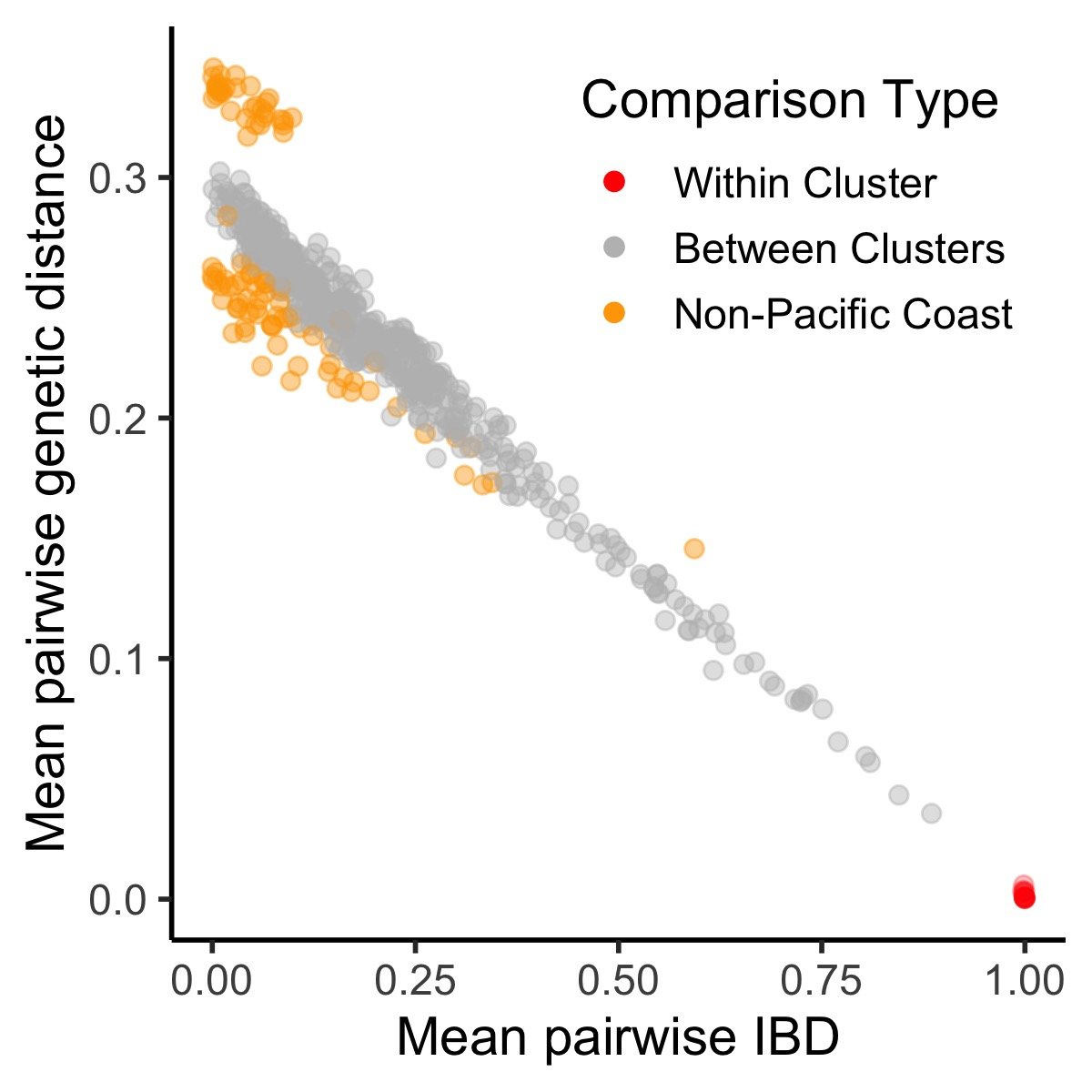

Supplement: S5 Fig — Mean genetic distance was calculated as the proportion of dissimilar calls within a set of 28,278 high-confidence SNPs. Both mean pairwise genetic distance and mean pairwise IBD were averaged over all inter-sample comparisons between each pair of clusters. Estimates for pairwise comparisons incorporating either of the two Venezuelan samples diverge to a greater extent, perhaps reflecting the inaccuracy of IBD estimation for samples originating outside the focal population. This is expected since population-level allele frequency estimates are required for IBD calculations, and these likely differ for the samples’ true population of origin. (TIF) [file ppat.1010993.s005.tif]

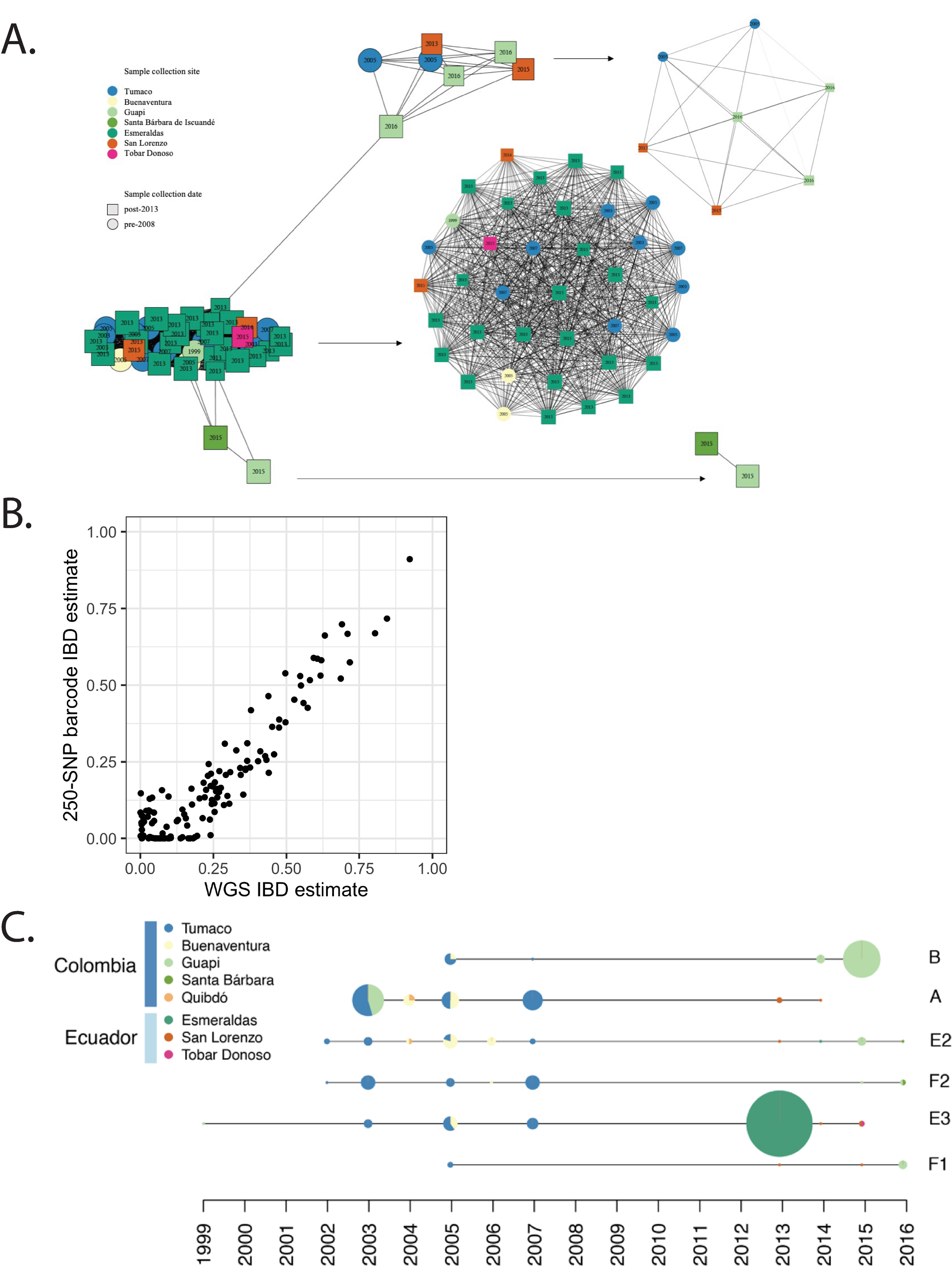

Supplement: S6 Fig — (A) In order to minimize cliques within components in the extended analysis, one clonal component that contained six cliques was broken down into three clusters: the largest containing two cliques, the other two continuing a single clique each. (B) IBD point estimates between clonal clusters were obtained with both full WGS data (x-axis) and using information from only 250 sites (y-axis). These estimates are highly correlated (Pearson’s r = 0.93). Values are calculated as the mean IBD of all between-cluster pairwise comparisons. (C) Exact sampling locations for clonal clusters depicted in Fig 4. (TIF) [file ppat.1010993.s006.tif]

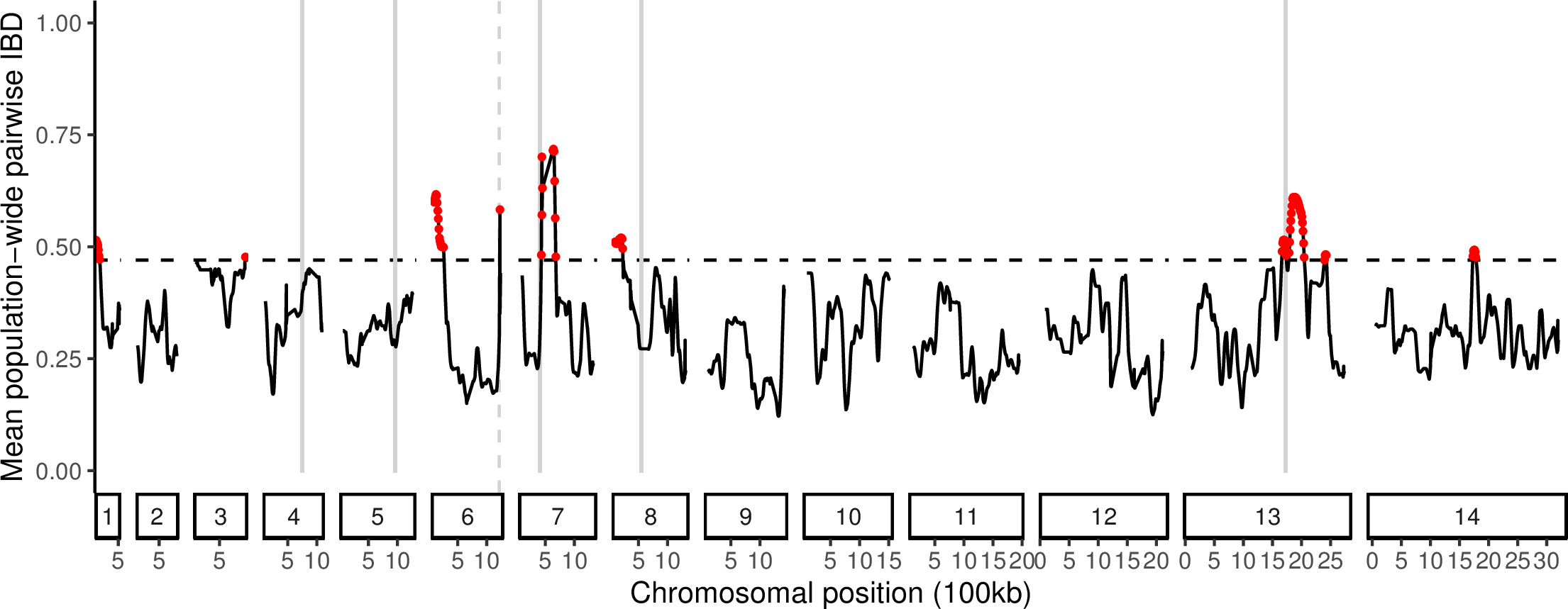

Supplement: S9 Fig — Each cluster was represented by the sample member with the most complete genome coverage. The dashed horizontal line marks the genome-wide 95th percentile. Windows falling above this threshold are marked with red points. Vertical lines mark known resistance loci: dhfr, mdr1, aat1, crt, dhps and kelch13. (TIF) [file ppat.1010993.s009.tif]

**A**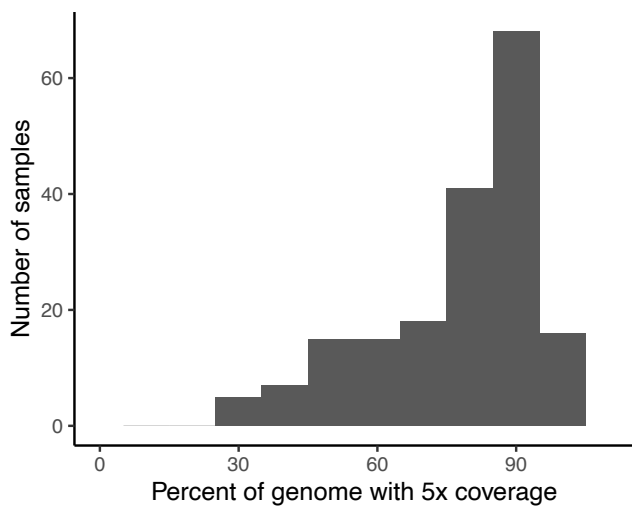**B**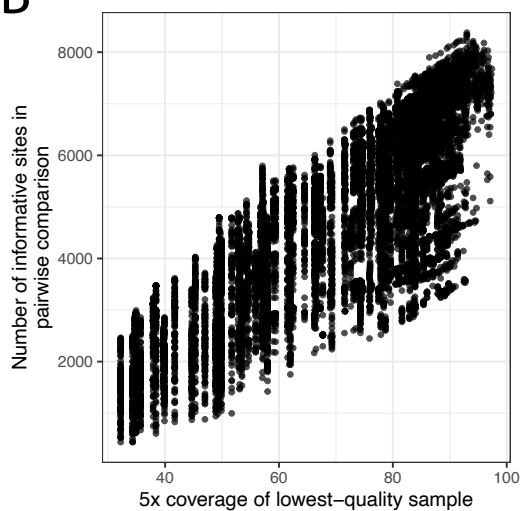**C**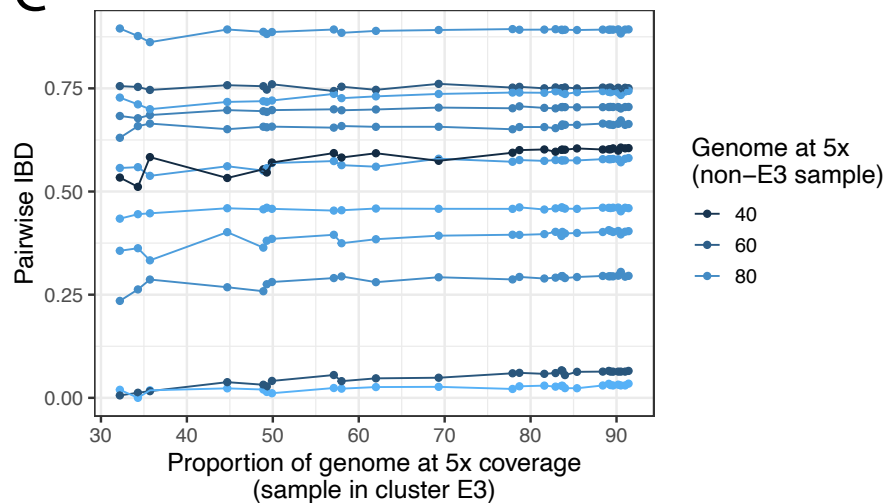**D**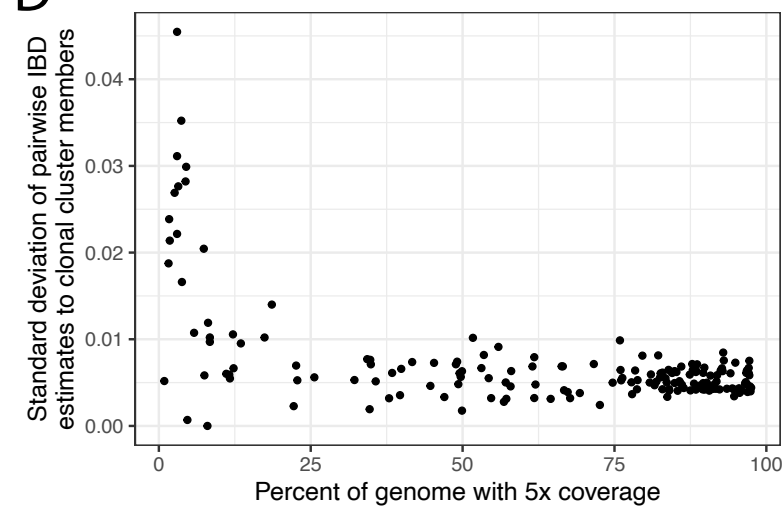

Supplement: S10 Fig — (A) WGS samples retained for downstream analysis varied in coverage with 30–97% of the genome being covered at 5x read depth. Median 5x coverage was 83%. (B) The number of informative sites reported by hmmIBD (y-axis) varied with sample coverage (x-axis). (C) Samples with 30% of the genome covered at 5x read depth performed comparably in hmmIBD to samples with high coverage. Depicted are the 23 samples within clonal cluster E3, plotted based on their genomic coverage (x-axis). Fractional IBD calculations were made for each E3 sample to 12 other samples that varied in relatedness (y-axis) and genome coverage (color). Naively, each comparator sample should have the exact same fractional IBD to all E3 samples as E3 sample genomes are clonal replicates. Overall, replicability of IBD estimates across E3 samples is high, with a slight deflation when coverage is low. (D) When samples with <30% of the genome at 5x were included in the analysis, artifacts and biases appeared in the data. These included spurious clique formation in the igraph analysis and an increase in the standard deviation among fractional IBD estimates made to members of the same clonal cluster. (PDF) [file ppat.1010993.s010.pdf]
