# Supplementary material for: Resolving drug selection and migration in an inbred South American Plasmodium falciparum population with identity-by-descent analysis
Source: PLoS Pathog. 2022 Dec 21;18(12):e1010993. doi: 10.1371/journal.ppat.1010993 (PMC9815574; doi:10.1371/journal.ppat.1010993)
Supplement: Attachment — Submitted filename: Response to Reviewer Comments.pdf [file ppat.1010993.s016.pdf]

Response to Reviewers

We thank the editor and reviewers for their careful reading of the text and helpful suggestions. Below we detail the changes we have made to the revised manuscript. In particular, we have:

- Added further details regarding our coverage cut-off. This includes new text in the Methods and a new Supplementary Figure (S10)
- Repeated our PCA and ADMIXTURE analyses with different variant filtration
- Modified our discussion of selection and effective population size
- Revised the text with an eye towards making it clear, accessible, and informative to a broad audience

~~~~~~

Both reviewers have mentioned the low threshold for genome wide coverage of 30% however I note that Table S1 indicates that most of the samples have high genome coverage. Please provide an additional supplementary figure showing the distribution of coverage values, and comment on how robust the measurements of IBD are for low coverage samples.

We understand these concerns and have provided more details in the text and a new multi-panel supplementary figure (S10) to illustrate how we arrived at this cutoff. Figure S10 A shows the overall distribution of coverage. Figure S10 B reports the distribution of the number of informative sites (as reported by hmmIBD) as a function of coverage.

IBD inference can be performed with a limited number of markers. For instance, IBD analyses have been implemented with SNP genotyping and amplicon panels comprising dozens to hundreds of markers (Tessema et al 2022; LaVerriere et al 2022; Taylor et al 2017). As shown in Supplementary Figure S6B, there is a high correlation between the fractional IBD estimates made with our WGS data and the fractional IBD estimates made with a 250-SNP panel. For the hmmIBD analysis of our WGS samples, we used 16,000 sites. A sample with 30% coverage would therefore have approximately 4,800 calls, a much higher density of variants than a SNP panel provides.

Illustrating that this level of coverage is sufficient, Sup Figure S10C shows the distribution of fractional IBD between each sample from clonal cluster E3 (which contains 3 samples with 5x coverage of 30-40%) and a representative set of other samples. In general, lower coverage leads to a slight deflation in IBD estimates, but none of our analyses relied on strict IBD point estimates that would be affected by this amount of error. Along this vein, it is worth noting that the lowest coverage samples (30-50% 5x coverage) are all members of larger clonal clusters, so any cluster-based analyses would have included information from higher coverage samples.

We did not go lower than this 30% threshold as we have found—both with this and other data sets—that overall data quality suffers and leads to data artifacts below this point (Early & Camponovo et al, 2022). As an illustration from this data set, S10 D shows the standard deviation of fractional IBD estimates made between a focal sample and all members of clonal
~~~~~~

cluster D (the largest cluster). The standard deviation markedly increases as 5x sample coverage drops below 25%. We also detected other artifacts–such as spurious clique formation in the igraph analysis–when including samples below, but not above, 30% coverage.

Reviewer's Responses to Questions

Part I - Summary

Please use this section to discuss strengths/weaknesses of study, novelty/significance, general execution and scholarship.

Reviewer #1: (No Response)

Reviewer #2: In this article, Carrasquilla and Early et al. present whole genome sequence data from 166 P. falciparum isolates collected predominantly from Colombia and Ecuador and estimate parasite relatedness within and between countries based on identity-by-descent. They also examine the prevalence of drug resistance mutations and their corresponding haplotypes to understand the selection for resistance in these populations. The sequencing data will be a valuable contribution to the field, although the analyses and conclusions are somewhat confirmatory, and in a few instances, overstated. Specific comments and questions are noted below.

Part II – Major Issues: Key Experiments Required for Acceptance

Reviewer #1: (No Response)

Reviewer #2: 1) Genome coverage of >=30% is quite low. Can the authors discuss how this threshold was selected, as prior studies have often used much higher cutoffs for genome coverage? Did the authors examine whether there was any clustering based on missingness, particularly in the PCA and ADMIXTURE analyses?

We empirically assessed the data to determine a coverage cutoff that maximizes sample inclusion and minimizes artifacts or bias. Both with this and an unrelated data set (Early & Camponovo, et al 2022) we have found that a reasonably conservative cutoff is 25-30% of the genome at 5x. As we detail at the beginning of our response, this is sufficient coverage for obtaining fractional IBD estimates that display minimal bias. Below this coverage level, we continue to see samples that look “good” and could likely be analyzed, but we also begin to observe artifacts and biases in the data, which is why we consider 30% to be relatively conservative.

In our response to the next comment, we address the impact of coverage on the PCA and ADMIXTURE analyses.

2) Can the authors clarify whether the same ~16K SNPs used to evaluate IBD from the WGS data were also used in the PCA and ADMIXTURE analyses? I believe ADMIXTURE assumes independent sites, which would necessitate LD-pruning to be performed prior to analysis. Was this done?

We thank the reviewer for this remark as it made us improve our analysis and its description in the methods section. For IBD, PCA and ADMIXTURE, we used different variant sets. Below, we clarify the ones used for PCA and ADMIXTURE. We have also added details regarding these analyses in the Methods section.

For PCA, we selected one representative sample from each IBD cluster, preferentially choosing the sample with the highest genome-wide coverage. We had not, however, done variant filtering based on missing data or linkage disequilibrium, which is essential for PCA as suggested by reviewer 2. We have now updated the analysis. We limit the analysis to one representative sample per IBD cluster with the highest coverage, and filter for biallelic variants present in 100% of samples which were subsequently LD-pruned. We used Plink for pruning with a squared correlation (r^2) of 0.1, sliding windows of 100 SNPs, and a step size of 10 SNPs. This reduced the number of variants included in the analysis to ~11K for the 23 individual samples. We have modified the figure accordingly and updated the Methods section. The new results are consistent with what we obtained previously: PC1 separates populations from the East and West of the Andes mountain range. PCA was not able to separate populations from Colombia and Ecuador with the first four principal components supporting the argument that these form a unique genetic unit different from Guyana.

As suggested, we have similarly updated the ADMIXTURE analysis by performing LD pruning using the same parameters described and now written in detail in the methods section. We have updated figures 3A and supplementary figures 3 and 4 to reflect the new results. With the LD-pruned data, we also obtain an optimal level of clusters of K=5. We have also repeated the same analysis but with the altered-frequencies and have updated the figure accordingly. We have described the interpretation of these results in the results section and figure legends.

Also, if the purpose of the ADMIXTURE analysis is to identify the number of genetic subpopulations, then highly related isolates (i.e., members of the same clonal lineage) would have to be removed, including only one member from each clonal cluster in the analysis. This would also negate the need to test different cluster frequencies.

If the purpose of the analysis was to determine if ADMIXTURE would identify the same clonal lineages identified using IBD estimates, then does it make sense to select K=5? The value of K was selected using the “elbow method’ based on the CV error plot (which is standard), but by design based on the value of K chosen for the analysis, the ADMIXTURE analysis would not identify all of the same clonal lineages. I suspect if K had been set to 17, the same clonal lineages would have been identified.

We have revisited the text to clarify the intent of the ADMIXTURE and NJ tree analyses. We wished to compare the approaches as they are typically performed to demonstrate the value added by an IBD approach. We therefore conducted the ADMIXTURE analysis using commonly employed best practices, as someone would do if they had no prior knowledge of relatedness in the population. By having the alternate analyses side-by-side, we hoped to aid the interpretation of the IBD results for those more familiar with seeing NJ trees or STRUCTURE plots, and then also show the added utility of calculating relatedness alone.

3) The authors have not analyzed how effective population size has changed over time, and thus, would not have evidence to conclude that drug resistance emerged under current demographic conditions, as stated in the conclusions. Particularly for older first line antimalarials, this selection likely took place decades ago, during a time when effective population size may have been higher than it is in the current sample set.

We appreciate the reviewer raising this issue, and we have revised wording throughout the manuscript to more carefully address this point. In particular, we take care to separate our discussion of selection for CQ and SP resistance (pre-1990 in Colombia) and our discussion of the release from SP pressure, which occurred after 2006 and so within the time period of our genomic analysis.

Part III – Minor Issues: Editorial and Data Presentation Modifications

Reviewer #1: This article very clearly demonstrates the use of relatedness analysis to infer population clusters that may be related to transmission events but also how this can help identify regions of the genome that are under selection from interventions, mainly drugs used against the malaria parasite. As malaria elimination programs drive down the prevalence of the disease, such analysis set the scene for future applications by malaria genomic surveillance programs. There are however minor issue that could help the wider audience better comprehend the data and approaches applied.

1. The data is significantly heterogeneous, including both temporal and spatial data but with a variety of marker sets used for different analyses. While the authors have carefully identified and justified the use of these, it could remain challenging to follow for many, especially those being newly introduced into this area. A supplementary table summarising the new and previous data could be helpful. In this context, not only 166 isolates are analysed as indicated in the supplement.

We thank the reviewers for this suggestion. We have now added a supplementary table that summarizes the four distinct data sets and lists which analyses included them. We have also clarified wording in places throughout the text.

2. The regional IBD is overrepresented by Columbia. At what level is IBD considered high and what statistics was applied for 0.36 between Colombia and Ecuador to be considered high?

There is no single threshold over which IBD is considered high. Here, we use the term in a relative sense, but to put the value in context, half-siblings are IBD across 25% of their genome (on average). We have added this contextualization in the Results section to clarify why a median IBD of 0.36 is considered highly inbred. As we show in Figure 2A, median IBD within other global populations—even other low transmission populations—is much lower than what we observe in the Pacific Coast Region.

IBD is in the core of the analysis, and this has been done with whole genome data and various SNP sets. As genome coverage could be as low as 30% and these were compared with those having higher coverage, the sets of markers across the ~16000 that are common to pairs of isolates could be summarised in supplements.

To address this, we have included panel B in our new supplementary figure (discussed more fully at the beginning of our response). This panel plots the number of informative sites (as identified by hmmIBD) as a function of coverage.

An extension of this heterogeneity is evident in the wide range used from SNP barcodes (12 to 248). The authors did indicate that they used the IBD CI to retain reliable IBD. What was the proportion of results retained from the low SNP numbers? This could inform the minimal number needed by those that would be applying this approach in resource limited settings.

We have added this information in the Discussion along with a note of caution since this threshold will vary with data sets:

> *With this data set, employing confidence intervals permitted the analysis of samples with as few as 42 SNP calls. We caution, however, that this number will vary among data sets—and even among samples—as the informativeness of sites depends on factors like population structure, linkage disequilibrium, and allele frequency.*

3. A significant amount of masking was done to reduce heterozygous calls and therefore artificially assigning monogenomes. An indication of the number of such sites masked and if they indeed passed quality filters for sequencing will be informative. Complex infections remain a challenge in higher transmission regions and managing this with new methods, rather than discarding them could be helpful. Notwithstanding, approximating clonality to 1 in this analysis enabled more reliable IBD estimates but the unfiltered data could help in better appreciating complexity for these populations.

Thank you for this comment as it showed that we had failed to include details about polyclonality assessment in the Methods. In fact, we did run TheRealMcCoil and then discarded samples that were likely polyclonal. The heterozygous-call masking was performed on the remaining monoclonal samples, as even monoclonal samples will have a small proportion of heterozygous calls due to genotyping error or low-level contamination.

We certainly agree that the field needs improved methods for leveraging data from complex infections. As we detail further in our response to Reviewer 2, we experimented with phasing

the genomes in the complex infections using DEploidIBD, but we didn't feel the results were compelling enough to be included in the manuscript.

4. The contribution of regions of selective sweeps to overall pairwise IBD could be biased. As these are selective signatures, isolates are more likely to be in IBD in these regions due to selection even though they may not be from the same lineage. The authors could discuss how this affected genome-wide pairwise IBD. As the variants included between pairs did vary, this may add to the heterogeneity as some pairs of isolates will have larger proportions of sweep variants being analysed.

IBD is indeed heterogeneous across the genome (Figure 5C). However, the physical distribution of calls across our low-coverage genomes is sufficiently broad to minimize any systematic bias due to selective sweeps. We believe this point is now illustrated by panel C in the new supplementary figure (detailed at the beginning of this response), which shows that 30-50% 5x genome coverage is sufficient for obtaining fractional IBD point estimates that have at most a small bias (<0.1).

5. The igraph plots in supplementary figures 2 and 7 could be placed in separate boxes or borders added to distinguish between sub-plots

We agree the current version of the plot is hard to read so we have revised Supplementary Figures 2 and 7 as suggested. For Supp. Figure 2 we have also scaled the figure so it would be easier to distinguish the colors.

Reviewer #2:

4) The results section includes substantial amounts of data interpretation that would normally be included in the discussion section.

We have revised the manuscript with this comment in mind. There still remain instances where we offer interpretation in the Results section, and this is intentional. We would like this manuscript to be accessible to a wide audience and so provide some interpretation and context in instances where we felt the analysis might be unfamiliar to a typical reader.

5) Minor: Although the number of polyclonal infections is relatively small, the predominant clone (if present) could be included in the analysis. As MOI is likely low in this setting (even if not equal to one), deconvolution of genomes using new tools may allow inclusion of genomes from these infections, rather than excluding them.

We agree with the utility of this suggestion, and have applied DEploidIBD to this data set to assess the accuracy of the algorithm for phasing polyclonal infections. Given the highly clonal structure in this population, the expectation is that the majority of polyclonal infections will carry at least one genome that was also characterized in a monoclonal infection. Using a nearest neighbor approach, we identified at least one previously characterized genome in 8 of 14 (57%) of the polyclonal infections. For these genomes, the mean sequence identity at variant sites was relatively high (92%), however, we did not think this was accurate enough to directly analyze the

inferred genomic sequences in the context of the IBD analysis, which was the central focus of this manuscript. Rather than publish this rough analysis, we plan to continue piloting other approaches and refining this analysis for future use so that polyclonal infections can be "rescued" for analysis.

6) Minor: In the drug resistance literature, the notation of single, double, and triple mutant (as noted in Figure 5c) is often not used for genes other than dhfr and dhps, e.g., pfcrt. This made interpretation of the figure challenging.

We agree with this point and for clarity, we have updated both Figure 5B and Supplementary Figure 7 to make interpretation of the drug-resistance haplotypes easier. We have denoted the drug resistance haplotypes with their amino-acid sequence at the relevant positions for all drug resistance genes described, rather than using the previous annotation (WT, single, double or triple).
